# Physical Comorbidity, Clinical Correlates, and Inpatient Management Burden in Hospitalized Patients With Major Depressive Disorder

**DOI:** 10.31083/AP51021

**Published:** 2026-10-14

**Authors:** Zhuo Jiang, Haining Song, Lingnan Gao, Chen Wei, Chenghao Zhang, Zhan Wang, Kun Li, Zhonggang Wang

**Affiliations:** ^1^Department of Psychiatry, Shandong Daizhuang Hospital, 272051 Jining, Shandong, China; ^2^Department of Psychiatry, Jining Medical University, 272000 Jining, Shandong, China; ^3^Department of Psychiatry, Zibo City Mental Health Center, 255100 Zibo, Shandong, China; ^4^School of Psychology, Qufu Normal University, 273165 Qufu, Shandong, China; ^5^Department of Physical Therapy, Shandong Daizhuang Hospital, 272051 Jining, Shandong, China

**Keywords:** major depressive disorder, physical comorbidity, comorbidity prevalence, clinical correlates, inpatient management burden

## Abstract

**Background:**

Major depressive disorder (MDD) is frequently accompanied by physical comorbidity, which may complicate inpatient assessment and management. However, large-scale patient-level studies investigating physical comorbidity and its impact on inpatient resource utilization among hospitalized patients with MDD remain limited. This study aimed to characterize the profile of physical comorbidity in MDD and examine its adjusted associations with indicators of inpatient management burden.

**Methods:**

In this large-scale cross-sectional study, we analyzed data from 8463 patients with MDD admitted to a major psychiatric center. Patients were stratified according to the presence or absence of physical comorbidity. We assessed the prevalence and spectrum of physical comorbidity, compared demographic and clinical characteristics between groups, and used multivariable regression models to identify factors associated with physical comorbidity, as well as to examine its associations with length of stay, internal medicine consultation, and the use of physical therapies.

**Results:**

Physical comorbidity was identified in 56.9% of hospitalized patients with MDD. Among those with physical comorbidity, 65.2% had physical multimorbidity (≥2 conditions). Diseases of the circulatory system (80.7%) and endocrine, nutritional, and metabolic diseases (35.4%) were the most common disease categories. Older age, farming occupation, and greater severity of depressive episodes were independently associated with physical comorbidity. Physical comorbidity was also associated with a longer length of stay and substantially higher odds of requiring internal medicine consultation.

**Conclusions:**

This large inpatient cohort demonstrates that physical comorbidity is common, multisystemic, and clinically relevant among hospitalized patients with MDD. These findings support the routine assessment of physical health and timely multidisciplinary care during hospitalization, particularly for older patients, farmers, and those with severe depressive episodes.

## Main Points

1. Physical comorbidity was common among patients with major depressive disorder (MDD), and many affected patients had multiple physical diseases across physiological systems.

2. Among patients with physical comorbidity, circulatory system diseases and endocrine, nutritional, and metabolic diseases were the predominant disease categories.

3. Physical comorbidity was associated with greater inpatient management burden, including longer length of stay and substantially increased odds of internal medicine consultation.

## 1. Introduction

Mental disorders constitute a major component of global disease burden, and depressive disorders account for a substantial share of this burden. Global Burden of Disease Study 2021 analyses indicate that mental disorders contribute substantially to non-fatal health loss worldwide [1]. In 2021, depressive disorders affected approximately 332 million people and accounted for more than 56 million disability-adjusted life years (DALYs) [2]. Major depressive disorder (MDD) can impair cognition, sleep, appetite, energy, daily functioning, family roles, and work or school performance, leading to persistent functional impairment and reduced quality of life [3]. Its burden also extends beyond psychological symptoms and social functioning. People with mental disorders experience marked physical health inequalities and premature mortality, and physical comorbidity is an important contributor to this mortality gap [4,5,6]. Understanding MDD from the perspective of physical health is therefore an important issue in depression research and psychiatric care.

This physical health perspective is clinically important because physical comorbidity constitutes a major part of the clinical burden of MDD [4,7,8]. This pattern can also be observed from the opposite clinical perspective. Depression has been reported in 29% of patients with myocardial infarction, 28% with type 2 diabetes, 23% with Parkinson disease, 18% with stroke, 16% with cancer, and 13% with Alzheimer disease, compared with an estimated MDD prevalence of approximately 5% in the general population [9]. In psychiatric care, physical comorbidity may increase the need for physical examination, laboratory monitoring, medication review, multidisciplinary consultation and assessment of drug-disease or drug-drug interactions, and is associated with greater healthcare resource use [4,7,8,10,11]. Thus, physical comorbidity is not only part of the clinical profile of MDD, but also a factor that shapes clinical assessment, treatment management, and healthcare resource burden.

Previous research has linked depression to physical disease burden across several settings. In populations with chronic illnesses, depression and depressive symptoms are highly prevalent across cardiometabolic, neurological, gastrointestinal, musculoskeletal, and respiratory categories [7,8,12]. Among people with depression, an outcome-wide multicohort study found associations with multiple physical diseases requiring hospital treatment, particularly endocrine and related internal organ diseases, musculoskeletal diseases, and circulatory and blood diseases [9]. A German nationwide ambulatory claims study also reported a broad comorbidity profile in patients with depression, with back pain, hypertensive diseases, and metabolic disorders among the most common physical diseases [13]. Longitudinal studies further suggest that depression is associated with subsequent physical multimorbidity accrual [14], and exhibits potential bidirectional relationships with cardiometabolic conditions [15]. Although physical comorbidity has been associated with greater healthcare resource use and longer hospital stays [10,16,17], studies specific to clinically diagnosed psychiatric inpatients with MDD remain limited. Few studies have jointly examined the prevalence, comorbidity count, system-level distribution, demographic and clinical correlates, and hospitalization-related indicators of physical comorbidity in this population, such as length of stay, internal medicine consultation, and treatment use.

To address this gap**, **the present study used patient-level routine inpatient data from a major psychiatric center to examine physical comorbidity among hospitalized patients with MDD. We described the prevalence of physical comorbidity, the number of non-psychiatric physical diseases, and their system-level distribution, identified associated demographic and clinical factors, and examined adjusted associations with length of stay, internal medicine consultation and selected non-pharmacological treatment-use indicators. By focusing on a large psychiatric inpatient cohort, this study provides setting-specific evidence for recognizing physical comorbidity and assessing its implications for inpatient management among hospitalized patients with MDD.

## 2. Materials and Methods

### 2.1 Study Design and Participants

This was a large-scale, cross-sectional study of retrospective data from the Hospital Information System at Shandong Daizhuang Hospital in China between January 1, 2024, and July 31, 2025. The initial extraction identified 9913 MDD-related hospitalization records during the study period. Diagnostic eligibility was determined using both admission and discharge diagnoses. Records were retained only when the admission diagnosis was MDD-related and the discharge diagnosis confirmed eligible MDD without exclusionary diagnoses. The inclusion criteria were as follows: (1) a primary diagnosis of MDD according to the International Classification of Diseases, 10th Revision (ICD-10) criteria, confirmed by two experienced psychiatrists [18]; (2) no specific restrictions on age; (3) Chinese nationality with no restrictions on ethnicity. Exclusion criteria were as follows: (1) a lifetime diagnosis of any other psychiatric disorder as defined by the ICD-10 criteria, including but not limited to schizophrenia, schizoaffective disorder, bipolar disorder, obsessive-compulsive disorder, or secondary depressive episodes attributable to medical conditions. (2) a lifetime diagnosis of intravenous drug dependence or active substance use disorders; (3) affective symptoms judged to be secondary to a general medical condition, medication use, or substance use. After diagnostic eligibility screening, 8822 eligible MDD-related hospitalization records remained. Five records with missing information on relevant variables were excluded. For patients with multiple eligible hospitalizations during the study period, only the first eligible hospitalization was retained as the index hospitalization. After excluding 354 subsequent hospitalization records, the final patient-level analytic sample comprised 8463 unique patients, with each patient contributing one index hospitalization. The sample selection and data-processing procedure is shown in Fig. 1.

**Fig. 1.**
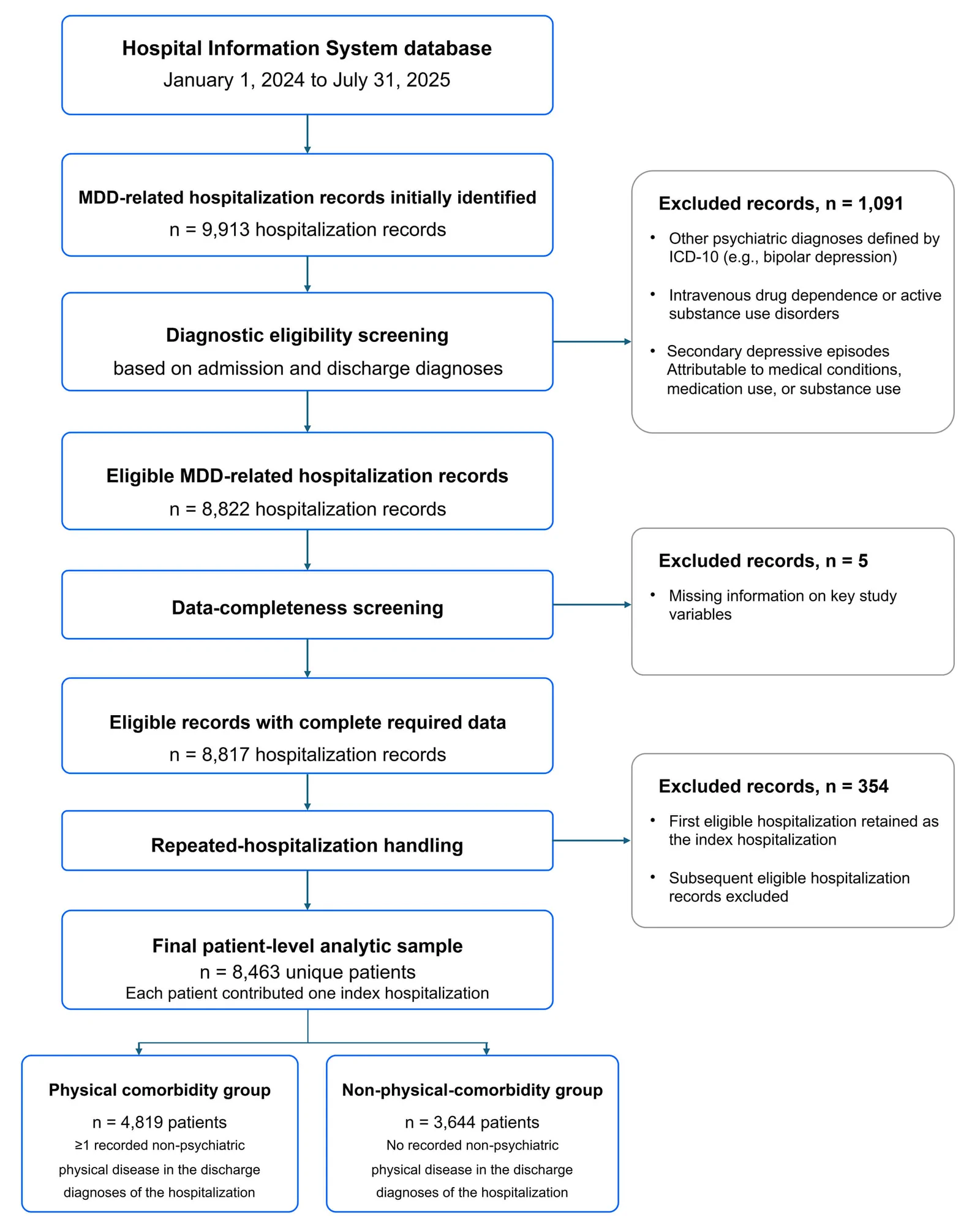
**Flow diagram of patient selection and construction of the patient-level analytic cohort**.

During the hospitalization, all participants underwent comprehensive laboratory tests and physical examinations. Physical comorbidity was defined as the presence of at least one non-psychiatric physical disease recorded in the discharge diagnoses of the index hospitalization. These diagnoses were made by attending physicians according to ICD-10 criteria. Based on the presence of physical comorbidity, patients were stratified into two groups: the comorbidity group and the non-comorbidity group. Non-psychiatric physical diseases were systematically categorized into 11 distinct physiological systems: circulatory system diseases; nervous system diseases; endocrine, nutritional, and metabolic diseases; digestive system diseases; respiratory system diseases; genitourinary system diseases; neoplasms; diseases of the blood and blood-forming organs; infectious diseases; musculoskeletal system diseases; and immune system diseases. Conditions not fitting these categories were classified as “other system diseases”. For patients in the comorbidity group, we further counted the number of comorbid conditions and the number of physiological systems involved, and summarized these distributions in chart form.

### 2.2 Assessment and Variables

Data were collected using structured self-designed questionnaires and extracted from the Hospital Information System. Variables were selected based on prior evidence regarding MDD, physical comorbidity, and healthcare resource use among inpatients, together with considerations of clinical relevance, data availability, and the actual clinical practice of the study hospital [3,7,10,19]. Demographic variables included: age (years), sex, ethnicity, educational level (years), occupational status (categorized as worker, farmer, student, or other), and marital status (unmarried, married, divorced, or widowed).

Clinical variables included age at onset, length of stay, family history of mental disorders, episode type, and the presence of psychotic features. Depressive episode severity was recorded as a diagnosis-based clinical classification of mild, moderate, or severe, as determined by the treating psychiatrists according to ICD-10 diagnostic guidelines based on the number of core and additional depressive symptoms and overall clinical impairment. Premorbid personality traits were categorized as introverted, extroverted, or ambivert. Treatment-related variables included internal medicine consultation, use of repetitive transcranial magnetic stimulation (rTMS), use of modified electroconvulsive therapy (MECT), and use of electroacupuncture therapy. These treatment variables were recorded as binary indicators of whether the intervention was used during hospitalization and were not intended to measure treatment response or comparative effectiveness. Lifestyle factors such as smoking and alcohol history were also recorded.

### 2.3 Statistical Analysis

All statistical analyses were performed using IBM SPSS Statistics, version 26.0 (IBM Corp., Armonk, NY, USA). A two-tailed *p* value < 0.05 was considered statistically significant.

Descriptive distributions of the number of comorbid conditions and affected physiological systems, as well as the frequency of comorbidities by system, were illustrated using bar charts. Given that continuous variables were not normally distributed, they are presented as median (interquartile range, IQR) and were compared between groups using the Mann–Whitney U test. Categorical variables are reported as frequencies and percentages and were compared using the chi-square test or Fisher’s exact test.

Binary logistic regression was used to identify factors associated with physical comorbidity. Age and sex were included a priori, and additional candidate predictors were selected based on clinical relevance and univariate comparisons; results were reported as regression coefficient (B), standard error (SE), *p*-value, odds ratios (ORs), and 95% confidence interval (CI). Model performance was evaluated using the omnibus test and Nagelkerke R^2^; the Hosmer–Lemeshow test was interpreted with caution given the large sample size.

To assess the association of physical comorbidity with healthcare utilization and treatment patterns during the index hospitalization, additional multivariable regression analyses were performed. A linear regression model with log-transformed length of stay was used, while logistic regression models were applied for binary treatment indicators. In the primary adjusted models, age, sex, occupation, and depressive episode severity were included as covariates. Given the marked age imbalance between the comorbidity and non-comorbidity groups, age-stratified models were further performed as an additional sensitivity analysis to evaluate whether the main associations were consistent across age strata. Age strata were defined according to quartiles of the overall age distribution: ≤22, 23–48, 49–62, and ≥63 years. The stratified models used the same covariate framework as the primary models, with age retained as a continuous covariate within each stratum where estimable. Regression coefficients and exponentiated estimates are reported where appropriate. For log-transformed continuous outcomes, exponentiated coefficients were interpreted as relative changes in the original outcome scale. 

## 3. Results

### 3.1 Demographic and Clinical Profile of the Study Population

A total of 8463 patients with MDD were included in the study. The overall demographic and clinical characteristics are summarized in Table 1. The median age was 48 (IQR 22–62) years. Females accounted for approximately two-thirds of the sample (66.58%). Most patients were married (59.45%) and of Han ethnicity (99.31%). The median education level was 9 (IQR 5–12) years. More than half of the patients (56.94%) had at least one comorbid physical disease.

**Table 1.** **Demographic and clinical characteristics of all participants studied**.

Variables		value
Age [years, M (P25, P75)]		48 (22, 62)
Sex [n (%)]		
	Male	2828 (33.42%)
	Female	5635 (66.58%)
Marriage [n (%)]		
	Unmarried	2736 (32.33%)
	Married	5031 (59.45%)
	Divorced	257 (3.04%)
	Widowed	439 (5.19%)
Ethnicity [n (%)]		
	Han	8405 (99.31%)
	Other	58 (0.69%)
Comorbidity [n (%)]		4819 (56.94%)
Non-comorbidity group [n (%)]		3644 (43.06%)
Education level [years, M (P25, P75)]		9 (5, 12)

Values are n (%) for categorical data and M (P25, P75) for continuous data. M, median; P25, 25th percentile; P75, 75th percentile.

### 3.2 Demographic Disparities Between Groups

The demographic profiles of patients with and without physical comorbidity diverged significantly (Table 2). Patients in the comorbidity group were markedly older than their non-comorbid counterparts, with a median age gap of 35 years (59 [IQR 45–68] years vs 24 [IQR 17–43] years; z = –51.75, *p* < 0.01). The comorbidity group was characterized by a higher prevalence of married (74.93%, n = 3611 vs 38.97%, n = 1420) and widowed (8.36%, n = 403 vs 0.99%, n = 36) individuals, whereas the non-comorbidity group consisted largely of unmarried patients (56.92%, n = 2074). Farmers were overrepresented in the comorbidity group (47.56%, n = 2292), which was also characterized by a lower educational level compared with the non-comorbidity group (z = –14.99, *p* < 0.01). In contrast, students comprised nearly one-third of the non-comorbidity group (30.95%, n = 1128).

**Table 2.** **Comparison of demographic characteristics between the two groups**.

		Comorbidity group (n = 4819, 56.94%)	Non-comorbidity group (n = 3644, 43.06%)	χ^2^/z -value	*p*-value
Age				–51.75	< 0.01**
	[years, M (P25, P75)]	59 (45, 68)	24 (17, 43)		
Sex [n (%)]				16.52	<0.01**
	Male	1523 (31.60%)	1305 (35.81%)		
	Female	3296 (68.40%)	2339 (64.19%)		
Marriage [n (%)]				1865.80	<0.01**
	Unmarried	662 (13.74%)	2074 (56.92%)		
	Married	3611 (74.93%)	1420 (38.97%)		
	Divorced	143 (2.97%)	114 (3.13%)		
	Widowed	403 (8.36%)	36 (0.99%)		
Occupation [n (%)]				1695.86	<0.01**
	Worker	949 (19.69%)	454 (12.46%)		
	Farmer	2292 (47.56%)	530 (14.54%)		
	Student	265 (5.50%)	1128 (30.95%)		
	Other	1313 (27.25%)	1532 (42.04%)		
Ethnicity [n (%)]				0.29	0.59
	Han	4788 (99.36%)	3617 (99.26%)		
	Other	31 (0.64%)	27 (0.74%)		
Education level				–14.99	< 0.01**
	[years, M (P25, P75)]	9 (5, 9)	9 (5, 12)		

**p < 0.01.

### 3.3 Clinical Presentation and Treatment Patterns

Compared with the non-comorbidity group (Table 3), the onset age of MDD was significantly later in the comorbidity group (z = –50.76, *p* < 0.01). This group also exhibited a more complex clinical and inpatient management profile, characterized by a higher proportion of recurrent episodes (χ^2^ = 301.10, *p* < 0.01), longer length of stay (z = –5.84, *p* < 0.01), and more frequent internal medicine consultation (χ^2^ = 477.92, *p* < 0.01). Regarding symptom profiles, psychotic features were less frequent in the comorbidity group (18.97%, n = 914 vs 29.36%, n = 1070), while premorbid personality traits differed between groups (χ^2^ = 50.87, *p* < 0.01), with a higher proportion of extroverted traits among comorbid patients.

**Table 3.** **Comparison of clinical characteristics between the two groups**.

		Comorbidity group (n = 4819, 56.94%)	Non-comorbidity group (n = 3644, 43.06%)	χ^2^/z -value	*p*-value
Age at onset				–50.76	< 0.01**
	[years, M (P25, P75)]	55.91 (39.81, 64.64)	21.74 (16.11, 39.88)		
Length of stay				–5.84	< 0.01**
	[day, M (P25, P75)]	30.00 (18.00, 42.00)	28.50 (14.00, 42.00)		
Family history				0.13	0.720
	Positive	721 (14.96%)	535 (14.68%)		
	Negative	4098 (85.04%)	3109 (85.32%)		
Episode type				301.10	<0.01**
	First episode	1943 (40.32%)	2163 (59.36%)		
	Recurrent	2876 (59.68%)	1481 (40.64%)		
Psychotic features				124.97	<0.01**
	Yes	914 (18.97%)	1070 (29.36%)		
	No	3905 (81.03%)	2574 (70.64%)		
Depressive episode severity				16.95	< 0.01**
	Mild	87 (1.81%)	52 (1.43%)
	Moderate	1205 (25.01%)	783 (21.49%)		
	Severe	3527 (73.19%)	2809 (77.09%)		
Premorbid personality				50.87	<0.01**
	Introverted	2706 (56.15%)	2323 (63.75%)		
	Extroverted	1697 (35.21%)	1040 (28.54%)		
	Ambivert	416 (8.63%)	281 (7.71%)		
Consultation				477.92	<0.01**
	Yes	2251 (46.71%)	859 (23.57%)		
	No	2568 (53.29%)	2785 (76.43%)		
rTMS				115.01	<0.01**
	Yes	3147 (65.30%)	2773 (76.10%)		
	No	1672 (34.70%)	871 (23.90%)		
MECT				20.87	<0.01**
	Yes	499 (10.35%)	495 (13.58%)		
	No	4320 (89.65%)	3149 (86.42%)		
Electroacupuncture				5.62	0.018*
	Yes	592 (12.28%)	387 (10.62%)		
	No	4227 (87.72%)	3257 (89.38%)		
Smoking				3.54	0.060
	Yes	1227 (25.46%)	994 (27.28%)		
	No	3592 (74.54%)	2650 (72.72%)		
Drinking alcohol				1.66	0.197
	Yes	1197 (24.84%)	950 (26.07%)		
	No	3622 (75.16%)	2694 (73.93%)		

*p < 0.05; **p < 0.01. rTMS, repetitive transcranial magnetic stimulation; MECT, modified electroconvulsive therapy.

Regarding treatment, patients with physical comorbidity received more frequent internal medicine consultations (46.71%, n = 2251 vs 23.57%, n = 859, χ^2^ = 477.92, *p* < 0.01), while rTMS (65.30%, n = 3147 vs 76.10%, n = 2773, χ^2^ = 115.01, *p* < 0.01) and MECT (10.35%, n = 499 vs 13.58%, n = 495, χ^2^ = 20.87, *p* < 0.01) were less frequently used. Notably, similar to MECT, electroacupuncture was infrequently used in both groups; however, its utilization was slightly higher in the comorbidity group (12.28%, n = 592 vs 10.62%, n = 387, χ^2^ = 5.62, *p* = 0.018).

### 3.4 Profile of Physical Comorbidity

Physical comorbidity in this cohort was characterized by high prevalence and systemic complexity. Among patients with physical diseases (n = 4819), the majority (65.2%) had physical multimorbidity (defined as ≥2 comorbidities), with 12.0% suffering from five or more concurrent diseases (Fig. 2). This complexity extended to physiological systems (Fig. 3): while disease burden was confined to a single system in 51.6% of cases, nearly half (48.4%) involved multisystem pathology. Regarding the disease spectrum among the 4819 comorbid patients (Fig. 4), circulatory system diseases predominated (80.7%), followed by endocrine, nutritional, and metabolic disorders (35.4%), digestive (15.3%), respiratory (12.3%), and nervous system diseases (11.2%).

**Fig. 2.**
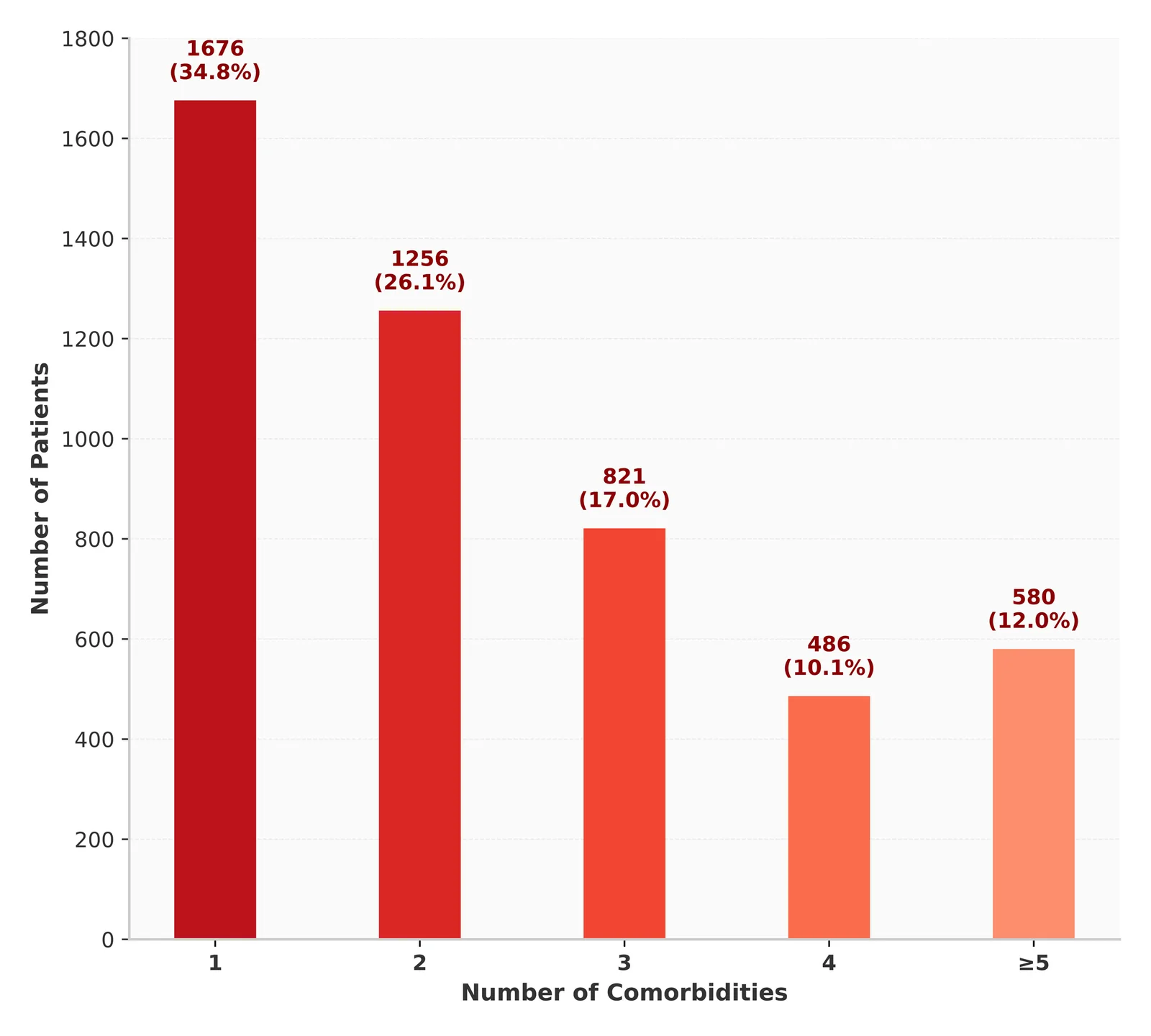
**Distribution of the number of physical diseases per patient with physical comorbidity**.

**Fig. 3.**
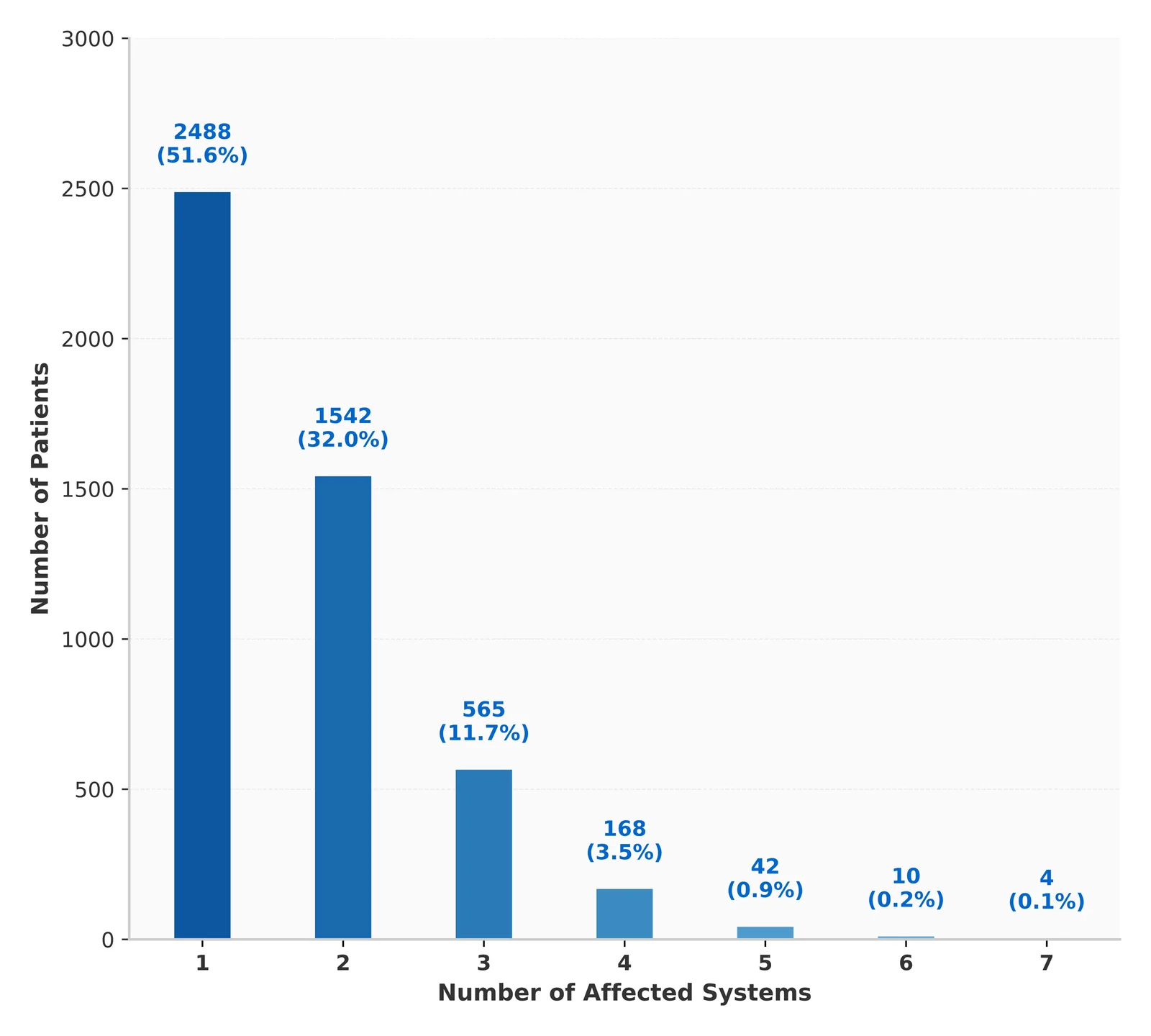
**Distribution of the number of physiological systems involved among patients with physical comorbidity**.

**Fig. 4.**
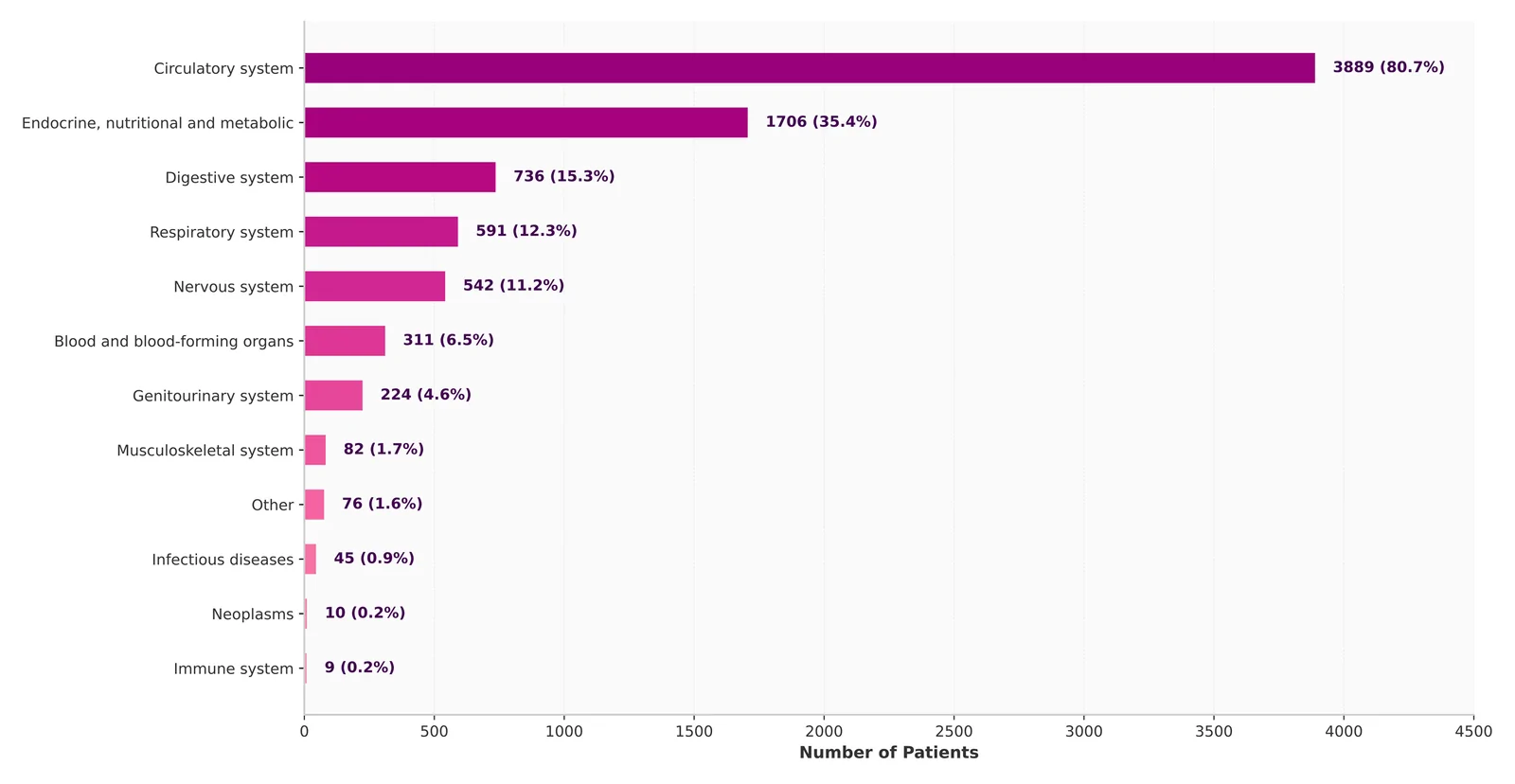
**System-level distribution of physical disease categories among patients with physical comorbidity**.

### 3.5 Correlates of Physical Comorbidity

Binary logistic regression identified several factors associated with physical comorbidity (Table 4). Older age was associated with higher odds of physical comorbidity (OR = 1.075, *p* < 0.01). Among occupational categories, being a farmer was associated with higher odds of physical comorbidity compared with the reference category (OR = 1.316, *p* < 0.01). Compared with patients with severe depressive episodes, those with mild (OR = 0.638, *p* = 0.026) and moderate depressive episodes (OR = 0.729, *p* < 0.01) had lower odds of physical comorbidity. A category-specific association was also observed for marital status, with married patients showing lower odds than widowed patients (OR = 0.646, *p* = 0.019).The model was statistically significant overall (Omnibus χ^2^ = 3080.710, *p* < 0.001; Nagelkerke R^2^ = 0.409).

**Table 4.** **Multivariable logistic regression analysis of factors associated with physical comorbidity in patients with major depressive disorder**.

		B	SE	*p*-value	OR	95% CI for OR	
						Lower	Upper
Age		0.072	0.003	<0.01**	1.075	1.069	1.081
Education level		–0.012	0.007	0.081	0.988	0.974	1.002
Sex							
	Male	0.091	0.058	0.118	1.095	0.977	1.228
	Female(ref.)	–	–	–	–	–	–
Marital status							
	Married	–0.437	0.186	0.019*	0.646	0.448	0.931
	Unmarried	0.015	0.220	0.944	1.016	0.660	1.562
	Divorced	–0.373	0.232	0.108	0.688	0.437	1.085
	Widowed(ref.)	–	–	–	–	–	–
Occupation							
	Worker	–0.108	0.087	0.212	0.898	0.758	1.063
	Farmer	0.274	0.080	<0.01**	1.316	1.125	1.539
	Student	–0.141	0.097	0.146	0.869	0.718	1.050
	Other(ref.)	–	–	–	–	–	–
Episode type							
	First-episode	–0.102	0.056	0.072	0.903	0.809	1.009
	Recurrent(ref.)	–	–	–	–	–	–
Psychotic Features							
	No	–0.120	0.067	0.073	0.887	0.778	1.011
	Yes(ref.)	–	–	–	–	–	–
Depressive episode severity							
	Mild	–0.450	0.202	0.026*	0.638	0.429	0.947
	Moderate	–0.316	0.069	<0.01**	0.729	0.637	0.834
	Severe(ref.)	–	–	–	–	–	–
Premorbid personality							
	Introverted	–0.049	0.102	0.630	0.952	0.781	1.162
	Extroverted	–0.139	0.107	0.194	0.870	0.706	1.073
	Ambivert(ref.)	–	–	–	–	–	–

Reference category (ref.); *p < 0.05; **p < 0.01. OR, odds ratio; CI, confidence interval. The model was fitted using binary logistic regression, with physical comorbidity status as the dependent variable. Model diagnostics: Omnibus χ^2^ = 3080.710, *p* < 0.001; Nagelkerke R^2^ = 0.409. Hosmer-Lemeshow χ^2^ = 23.543, *p* = 0.003.

### 3.6 Secondary Analyses of Healthcare Utilization and Clinical Management

After adjustment for age, sex, occupation, and severity of depressive episodes (Table 5), physical comorbidity was associated with approximately 31% longer length of stay. Furthermore, the presence of physical comorbidity was strongly associated with an increased likelihood of receiving internal medicine consultation (OR = 3.395, *p* < 0.01). In the overall adjusted models, physical comorbidity was not independently associated with electroacupuncture use, but was associated with slightly higher odds of MECT use (OR = 1.331, *p* < 0.01) and lower odds of rTMS use (OR = 0.856, *p* < 0.01). However, age-stratified sensitivity analyses based on quartiles of the overall age distribution showed that these associations were not consistent across age strata (**Supplementary Table 1)**. Age-stratified sensitivity analyses showed that the association with internal medicine consultation remained relatively stable across age strata, whereas the association with length of stay was generally consistent in direction but attenuated in the ≥63-year stratum (**Supplementary Table 1**). For selected non-pharmacological treatment-use indicators, the rTMS association was mainly observed in the ≤22-year and 23–48-year strata, whereas the MECT association was strongest in the ≤22-year stratum and was weaker but still statistically significant in the 23–48-year stratum.

**Table 5.** **Adjusted associations between physical comorbidity and healthcare utilization or treatment-use outcomes in patients with MDD**.

	Model	B value	OR/exp(B)	*p*-value	95% CI	
					Lower	Upper
Length of stay	ln (LOS), linear regression	0.271	1.311	<0.01**	1.250	1.375
internal medicine consultation	Logistic regression	1.222	3.395	<0.01**	3.022	3.813
Electroacupuncture	Logistic regression	–0.043	0.958	0.612	0.813	1.130
MECT	Logistic regression	0.286	1.331	<0.01**	1.135	1.562
rTMS	Logistic regression	–0.156	0.856	<0.01**	0.761	0.962

**p < 0.01, For continuous outcomes (length of stay), linear regression with log-transformation was applied. Results are reported as regression coefficient (B) on the natural log scale and exponentiated values [exp (B)]. LOS, length of stay.

## 4. Discussion

In this large patient-level inpatient cohort, physical comorbidity was highly prevalent among hospitalized patients with MDD, correlating with greater inpatient management complexity. More than half of the patients had at least one non-psychiatric physical disease, with multimorbidity and multisystem involvement being notably frequent. Circulatory system diseases, endocrine, nutritional, and metabolic diseases were the main comorbidity types**. **Furthermore, older age, farming occupation, and greater diagnosis-based depressive episode severity were independently associated with physical comorbidity. Physical comorbidity was also associated with longer hospitalization, increased demand for internal medicine consultations, and different patterns of rTMS and MECT use. These findings suggest that physical comorbidity is not merely an additional diagnosis in hospitalized patients with MDD. Rather, it is closely related to inpatient management complexity.

The findings are broadly consistent with previous evidence on physical comorbidity in depression, while adding a more specific psychiatric inpatient perspective. Prior reviews, cohort studies, and database analyses have shown that MDD is associated with cardiovascular, metabolic, neurological, gastrointestinal, musculoskeletal, and other chronic physical diseases [7,8,9,20,21,22,23]. Evidence from broader mental-health populations suggests that physical comorbidity is associated with greater healthcare resource use [10,13]. However, much of the existing evidence has been derived from general populations, outpatient data, or cohorts defined by physical diseases. By contrast, this study focused on clinically diagnosed inpatients with MDD and jointly examined the physical comorbidity profile, length of stay, internal medicine consultation, and selected non-pharmacological treatment-use indicators.

In this inpatient cohort, circulatory system diseases and endocrine, nutritional, and metabolic diseases were the leading comorbidity categories. This profile aligns with previous evidence indicating that physical comorbidity in depression often involves chronic physical diseases, such as cardiovascular and endocrine diseases [15,24,25,26,27,28,29]. Previous research suggests a potential bidirectional relationship between depression and these highly prevalent disease categories, which may involve shared behavioral and biological processes, including lifestyle disruption, neuroendocrine dysregulation, inflammation, and metabolic abnormalities [30,31,32,33,34,35,36,37]. However, given the cross-sectional design and the absence of biomarker data in our study, we can ascertain neither the causal direction nor the underlying mechanisms. Consequently, these shared pathways serve strictly as a theoretical framework for interpreting the observed comorbidity profile, rather than as empirical conclusions verified by our findings.

Within this comorbidity pattern, we identified several demographic and clinical characteristics associated with a higher likelihood of physical comorbidity. Age was one of the most stable correlates of physical comorbidity. The marked age difference between patients with and without physical comorbidity indicates that age was both a covariate requiring adjustment and an important clinical context for interpretation. This finding is clinically plausible, as chronic physical disease burden generally accumulates with age, and older patients with MDD may be more likely to have physical multimorbidity [38,39]. Farmers demonstrated significantly elevated odds of physical comorbidity compared with other occupational groups. Although previous research suggests that farming populations may experience a higher burden of mental health problems than some non-farming occupational groups [40], the specific link between farming occupation and physical comorbidity in patients with MDD remains underexplored. Therefore, this finding should be interpreted cautiously, as it likely reflects a complex interplay of socioeconomic determinants, rural living conditions, healthcare accessibility, and chronic disease management [40,41,42]. Furthermore, given the substantial cross-national heterogeneity in these structural factors among agricultural populations, this conclusion may not be readily generalizable to other countries. Despite this geographical limitation, both previous research and the present findings suggest that farming occupation may serve as a practical clinical flag for identifying patients who require proactive physical-health screening. Future studies should clarify the specific social and healthcare-related pathways driving this occupational disparity. Similarly, greater depressive episode severity was likewise associated with physical comorbidity. This finding is consistent with previous evidence suggesting that depression severity and symptom complexity may be related to the accumulation of physical conditions [43], and it indicates that severe MDD may represent not only higher psychiatric symptom burden but also a more complex overall health state and greater need for physical-health monitoring [3,7,33,44]. Future studies incorporating socioeconomic metrics and standardized depression scales are required to further elucidate these relationships.

Beyond patient characteristics, the resource-use results were more consistent. One of the most clinically relevant findings is the association between physical comorbidity and greater inpatient resource use. After adjustment for age, sex, occupation, and depressive episode severity, physical comorbidity remained associated with approximately 31% longer hospital stay. Patients with physical comorbidity also had substantially higher odds of internal medicine consultation. The age-stratified sensitivity analyses provided further context for these findings. The association with internal medicine consultation remained significant across all age strata, suggesting that this finding was not explained solely by the age imbalance between groups. Conversely, the associations with length of stay were not statistically significant in the oldest age stratum (≥63 years). In geriatric populations, length of stay may be influenced by factors beyond recorded physical comorbidity, including functional impairment, multimorbidity severity, and medication burden [45]. Consequently, this profound age-related burden may overshadow the additional resource demands of specific physical comorbidities [39,43]. Previous studies have shown that physical comorbidity in people with mental disorders is associated with greater healthcare resource use [10,16,17], and our findings extend this evidence to psychiatric inpatients with MDD. The persistence of the consultation finding in age-stratified analyses further suggests that physical comorbidity is closely related to medical assessment, consultation demand, treatment coordination, and service planning during hospitalization. These results support structured physical-health assessment and timely multidisciplinary collaboration, including assessment of cardiovascular and metabolic conditions, essential medical testing, medication review, and internal medicine consultation when clinically indicated [4,19,46]. Such integrated care may be particularly important when older age, farming occupation, and severe depressive episodes coexist.

Regarding selected non-pharmacological treatments, the overall adjusted model showed that physical comorbidity was associated with lower odds of rTMS use and slightly higher odds of MECT use, whereas electroacupuncture showed no independent association. However, the age-stratified analyses did not consistently support these overall treatment-use findings. The rTMS association was mainly observed in the lowest age stratum (≤22 years) and the lower age stratum (23–48 years). The MECT association was largely confined to the lowest age stratum (≤22 years). These discrepancies suggest that the treatment-use findings should be interpreted narrowly. They may reflect the combined influence of age structure, depressive severity, contraindication assessment, medical complexity, and clinical selection factors [47,48,49,50,51]. However, this dataset did not include treatment indications, contraindications, treatment parameters, clinical response, or adverse events. Therefore, the observed associations should be interpreted only as differences in treatment use during hospitalization, not as evidence of treatment suitability, effectiveness, or safety. The absence of an independent association with electroacupuncture may reflect institutional treatment pathways, patient acceptance, or its adjunctive role rather than physical comorbidity status itself [52].

Several strengths and interpretive boundaries should be considered. This study used a large psychiatric inpatient sample and patient-level data, reducing the influence of repeated admissions and allowing physical comorbidity to be examined together with clinical characteristics and inpatient management indicators. This design links epidemiological description with routine psychiatric inpatient care. However, because the study was single-center, retrospective, cross-sectional, and based on routine discharge diagnoses, the findings should be interpreted as evidence of inpatient clinical characteristics and management burden rather than as evidence of causal mechanisms or treatment effects.

Overall, this study shows that physical comorbidity is common and often multisystemic among hospitalized patients with MDD, and that it is closely related to inpatient management needs. These findings support greater attention to physical-health assessment and multidisciplinary coordination in psychiatric inpatient care, particularly for older patients, farmers, and patients with more severe depressive episodes. Future multicenter prospective studies should incorporate physical disease severity and control status, medication exposure, socioeconomic factors, biomarkers, and treatment outcomes to clarify temporal relationships, potential mechanisms, and integrated-care pathways for physical comorbidity in MDD.

### Limitations

Several limitations should be noted. First, the cross-sectional design precludes causal inference regarding the relationships between MDD and physical comorbidity. Second, physical comorbidity was based on routine clinical diagnoses recorded in the hospital information system. Although patients underwent routine physical examinations and laboratory testing during hospitalization, detailed information on physical disease duration, severity, and control status was not available. Third, the dataset did not include detailed medication information, including dose, duration, adherence, antipsychotic exposure, or medications used for physical diseases. Health insurance type, lifestyle factors, socioeconomic status, and healthcare-accessibility indicators were also unavailable or incomplete, leaving the possibility of residual confounding. Fourth, rTMS, MECT, and electroacupuncture were recorded only as binary treatment-use indicators. Treatment indications, contraindications, stimulation or procedural parameters, clinical response, and adverse events were not captured. Therefore, these findings should be interpreted as descriptive inpatient treatment-use patterns rather than evidence of treatment suitability or effectiveness. Finally, this was a single-center inpatient study, which may limit generalizability to patients with milder depression, outpatient or primary-care populations, and other healthcare systems.

## 5. Conclusions

This large patient-level inpatient study shows that physical comorbidity is common and often multisystemic among hospitalized patients with MDD. Circulatory system diseases and endocrine, nutritional, and metabolic diseases were the main comorbidity types. Older age, farming occupation, and greater depressive episode severity were associated with physical comorbidity, while physical comorbidity was related to greater inpatient management complexity and resource use among hospitalized patients with MDD. These findings indicate the need for timely and comprehensive physical-health screening during hospitalization, particularly for older patients, farmers, and patients with severe depressive episodes. Future research should adopt prospective longitudinal designs and integrate neuroimmune and metabolic biomarkers to clarify temporal relationships and potential bidirectional mechanisms. Subsequent studies should also incorporate granular socioeconomic phenotyping and geospatial healthcare-accessibility metrics to better clarify the pathways underlying the observed occupational vulnerabilities in farming populations, ultimately informing targeted multidisciplinary care models.

## Data Availability

The datasets generated and analyzed during the current study are not publicly available because they were derived from the hospital information system and contain sensitive patient information. De‑identified data may be available from the corresponding authors on reasonable request and following approval by the institutional ethics committee and data‑governance bodies.
