# Supplementary material for: Physical Comorbidity, Clinical Correlates, and Inpatient Management Burden in Hospitalized Patients With Major Depressive Disorder
Source: Alpha Psychiatry. 2026 Oct 14;27(5):51021. doi: 10.31083/AP51021 (PMC13731990; doi:10.31083/AP51021)
Supplement: Supplementary file 1 [file 2757-8038-27-5-51021-s1.zip › Supplementary Table 1.docx]

**Supplementary Table 1**

| **Outcome** | **Age stratum** | **Model N** | **Comorbid n** | **Non-comorbid n** | **B** | **Exp(B)/OR** | **95% CI** | **P value** |
| --- | --- | --- | --- | --- | --- | --- | --- | --- |
| Length of stay | Q1: ≤22 years | 2213 | 464 | 1749 | 0.310 | 1.36 | 1.23–1.51 | <0.001 |
| Length of stay | Q2: 23–48 years | 2020 | 869 | 1151 | 0.314 | 1.37 | 1.25–1.50 | <0.001 |
| Length of stay | Q3: 49–62 years | 2226 | 1635 | 591 | 0.208 | 1.23 | 1.14–1.33 | <0.001 |
| Length of stay | Q4: ≥63 years | 2004 | 1851 | 153 | 0.076 | 1.08 | 0.95–1.23 | 0.251 |
| Internal medicine consultation | Q1: ≤22 years | 2213 | 464 | 1749 | 1.286 | 3.62 | 2.92–4.48 | <0.001 |
| Internal medicine consultation | Q2: 23–48 years | 2020 | 869 | 1151 | 1.286 | 3.62 | 2.97–4.41 | <0.001 |
| Internal medicine consultation | Q3: 49–62 years | 2226 | 1635 | 591 | 1.066 | 2.90 | 2.34–3.60 | <0.001 |
| Internal medicine consultation | Q4: ≥63 years | 2004 | 1851 | 153 | 1.092 | 2.98 | 1.99–4.46 | <0.001 |
| Electroacupuncture | Q1: ≤22 years | 2213 | 464 | 1749 | -0.062 | 0.94 | 0.64–1.38 | 0.751 |
| Electroacupuncture | Q2: 23–48 years | 2020 | 869 | 1151 | 0.078 | 1.08 | 0.82–1.43 | 0.588 |
| Electroacupuncture | Q3: 49–62 years | 2226 | 1635 | 591 | -0.241 | 0.79 | 0.61–1.01 | 0.061 |
| Electroacupuncture | Q4: ≥63 years | 2004 | 1851 | 153 | 0.335 | 1.40 | 0.77–2.54 | 0.271 |
| MECT | Q1: ≤22 years | 2213 | 464 | 1749 | 0.488 | 1.63 | 1.24–2.14 | <0.001 |
| MECT | Q2: 23–48 years | 2020 | 869 | 1151 | 0.232 | 1.26 | 1.00–1.59 | 0.048 |
| MECT | Q3: 49–62 years | 2226 | 1635 | 591 | 0.106 | 1.11 | 0.80–1.54 | 0.528 |
| MECT | Q4: ≥63 years | 2004 | 1851 | 153 | -0.351 | 0.70 | 0.26–1.87 | 0.481 |
| rTMS | Q1: ≤22 years | 2213 | 464 | 1749 | -0.327 | 0.72 | 0.56–0.93 | 0.011 |
| rTMS | Q2: 23–48 years | 2020 | 869 | 1151 | -0.241 | 0.79 | 0.65–0.95 | 0.015 |
| rTMS | Q3: 49–62 years | 2226 | 1635 | 591 | -0.040 | 0.96 | 0.78–1.18 | 0.703 |
| rTMS | Q4: ≥63 years | 2004 | 1851 | 153 | 0.037 | 1.04 | 0.74–1.46 | 0.832 |
